# Nanomaterials as a Successor of Antibiotics in Antibiotic-Resistant, Biofilm Infected Wounds?

**DOI:** 10.3390/antibiotics10080941

**Published:** 2021-08-04

**Authors:** Marcela Nowak, Wioletta Barańska-Rybak

**Affiliations:** Clinic of Dermatology, Venereology and Allergology, Medical University of Gdansk, 80-210 Gdańsk, Poland; Wioletta.baranska-rybak@gumed.edu.pl

**Keywords:** nanomaterials, biofilm, wound management

## Abstract

Chronic wounds are a growing problem for both society and patients. They generate huge costs for treatment and reduce the quality of life of patients. The greatest challenge when treating a chronic wound is prolonged infection, which is commonly caused by biofilm. Biofilm makes bacteria resistant to individuals’ immune systems and conventional treatment. As a result, new treatment options, including nanomaterials, are being tested and implemented. Nanomaterials are particles with at least one dimension between 1 and 100 nM. Lipids, liposomes, cellulose, silica and metal can be carriers of nanomaterials. This review’s aim is to describe in detail the mode of action of those molecules that have been proven to have antimicrobial effects on biofilm and therefore help to eradicate bacteria from chronic wounds. Nanoparticles seem to be a promising treatment option for infection management, which is essential for the final stage of wound healing, which is complete wound closure.

## 1. Introduction

### 1.1. Background Information

Chronic wounds are a huge burden, both for affected patients with pain and reduced quality of life and for society. They are a major challenge for healthcare, as chronic wounds stand for significant resource needs and costs for treatment. The European Wound Management Association (EWMA) distinguishes between three main categories, namely 1. initial costs for assessment of the wound; 2. treatment of the wound and 3. care and treatment of the wound. Ragnarson et al. discovered that the weekly cost for treatment of patients with venous leg ulcers in Sweden in 2004 was estimated at between SEK 600 and 1400, depending on the size of the wound, and that the annual direct costs were between SEK 17,000 and SEK 26,500 per patient [1]. The treatment cost includes personnel resources and materials for wound dressing, of which between 65 and 69 percent of costs consist of the wound dressing itself. Guest et al. has estimated that the cost of treatment in the UK per patient in 2012–2013 was between GBP 788 (healed wounds) and GBP 4772 (unhealed wounds) per year to treat the wound and related diseases [2]. This represented 4% of the total expenditure on publicly funded healthcare in the UK in 2013.

The economic aspect of wound management is not only a major problem in European countries, but also worldwide. Sen et al. estimated that costs in the United States were approximately USD 50 billion per year for treatment of wounds, equivalent to 5% of their total annual spending on both Medicare and Medicaid, combined with USD 25 billion annually for the treatment of chronic wounds [3]. In addition, medical expenses in the United States associated with venous ulcers in 2007 were estimated at between USD 6391–7086 per patient and per year; in addition, these patients had a greater rate of absence from work than the control group.

The majority of chronic wounds do not heal due to a secondary infection, which alters the repair process. Moreover, most of the wounds are infected with bacteria that are resistant to commonly used antibiotics. WHO estimated that around 500,000 people worldwide are infected with multiresistant bacteria [4,5].

### 1.2. Biofilm

Prolonged healing is caused by uncontrolled bacterial growth supported by the prevalence of biofilms, which protect integral bacteria. Biofilm consists of microorganisms coated with a self-produced protective extracellular matrix [6]. This is usually made of a mix of bacteria, fungi, algae, yeasts, microbes and cellular debris. Biofilm formation is typically clinically found in patients with nonhealing infections, such as chronic infected wounds, osteomyelitis, chronic otitis media, chronic rhinosinusitis, recurring urinary tract infections, endocarditis, lung infections due to cystic fibrosis and patients with all sorts of foreign bodies including prosthetic implants [7]. All of these infections heal slower or do not heal at all, even if treated with standard antibiotics. Notable for infected wounds is that they tend to be more sloughy and have an unpleasant odour.

**Figure 1:**
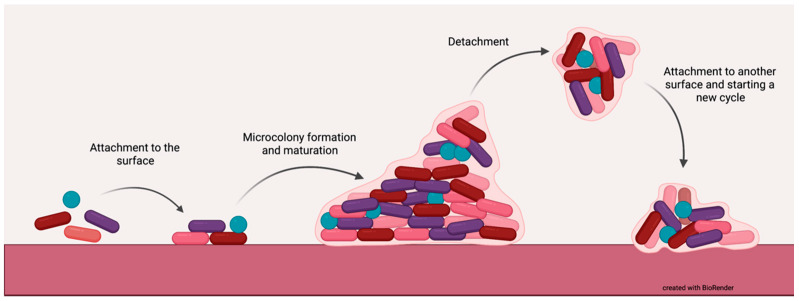
Cycle of biofilm formation.

Biofilm allows bacteria to survive in hostile conditions, therefore making them resistant to antimicrobials and immune system response, leading to prolonged infections and nonhealing wounds [8]. Bacteria within biofilm are notable for their adaptation skills, as they can withstand anoxia and nutrient limitation by altering gene expression and protein production of metabolism determining substances, therefore inhibiting metabolic rate and reducing the rate of cell division. Biofilm formation itself is divided into four stages: 1. irreversible bacterial attachment to the tissue surface; 2. microcolony formation; 3. biofilm maturation and 4. detachment or dispersion, which allows biofilm colonies to invade other areas [9] [Figure 1]. 

It is estimated that 60–80% of chronic infections treated in hospitals are caused by biofilm abundance [10]. Biofilm infected wounds are difficult to treat as they are commonly resistant to conventional antibiotic treatment, which explains the urgency for the development of new, more effective treatment options. Until today, most of the infections caused by biofilm have been treated with a wide spectrum of antibiotics. The biggest challenge of this type of treatment is that low doses are ineffective, whereas high doses might be toxic. The main modes of action of antibiotics are namely disruption of cell wall synthesis, translation and DNA replication of bacteria. Bacteria have developed resistance mechanisms that enable them to degrade antibiotics by enzymes such as β-lactamases, acetyltransferases or aminoglycoside modifying enzymes or by efflux pumps that may result in multidrug resistance. Nowadays, almost all bacteria have developed resistance against all antibiotics that are in use. Furthermore, no new antibiotics have been discovered in the past decade. Some enzymes such as proteases, DNAse, alginate lyase, amylase and cellulase have been reported to hasten the biofilm detachment, therefore disinfecting agents containing those enzymes seem to be a promising option of treatment of chronic wounds. Innovative biofilm eradication methods are constantly tested and include application of antibiofilm nanoparticles (NPs) [11,12]. NPs are promising as their mode of action is different that antibiotics, since it relies mostly on direct contact, therefore it is considered less possible that bacteria will develop resistance towards them.

### 1.3. Nanoparticles

NPs’ applications have been studied for over 20 years. Lipids, liposomes, cellulose, silica and metal can be carriers of NPs. What is more, NMs have been known since ancient times, although without detailed knowledge of their properties [13]. There are a few examples from classical antiquity potters and glassmakers, such as the Roman Lycurgus cup of dichroic glass from 4th century CE or silver pottery from Mesopotamia from 9th century CE, where silver and copper NPs were dispersed in glassy glaze. Furthermore, NPs can be found in nature, as they are components of atmospheric pollution and are ingredients in paints, plastics, metals, ceramics and magnetic articles. The field that studies NPs is called nanotechnology and Michael Faraday is the key grounder of it. In 1857 he was the first one to describe NPs and their optical properties. The 1970s and 1980s were fundamental years for studies on NPs, then called ‘ultrafine particles’. 

Oddly, the properties of NPs can differ very much from the same bulk materials before their division. Their properties have been scrupulously researched and are mostly owing to their large area–volume ratio [13]. The high surface area allows heat, ions and molecules to diffuse into the particles at a higher and faster rate. When dissolved in a different medium, the interfacial layer can change the chemical and physical properties of NPs. This layer is often considered as an inseparable part of NPs and it is thought to be one of the main reasons for their activity. The interaction between NPs’ surface and solvent also makes it easier for NPs to gather into suspensions and avoid floating. NPs usually contain core molecules and a shell that stabilizes the NPs and aids their function by preventing their degradation, oxidation and by increasing their biocompatibility. 

Moreover, core–shell NPs may gain both electric and magnetic properties, different from their bulk derivatives, by upconverting and downconverting NPs and a shift in different wavelength spectrum emission [14]. Core–shell NPs produced from two different metals result in the formation of a core–shell structure where completely new properties are found. 

NPs have been proven to possess possible dangers both to the environment and individuals treated, which are mostly caused by their high surface to volume ratio that boosts their catalytic activity. Furthermore, they attach and aggregate on the phospholipid layer and pass through the membrane. Their interaction within the cells remains unknown, yet it is unlikely that they might cross cell nucleus, Golgi apparatus or endoplasmic reticulum owing to particle size and aggregation susceptibility. 

**Figure 2:**
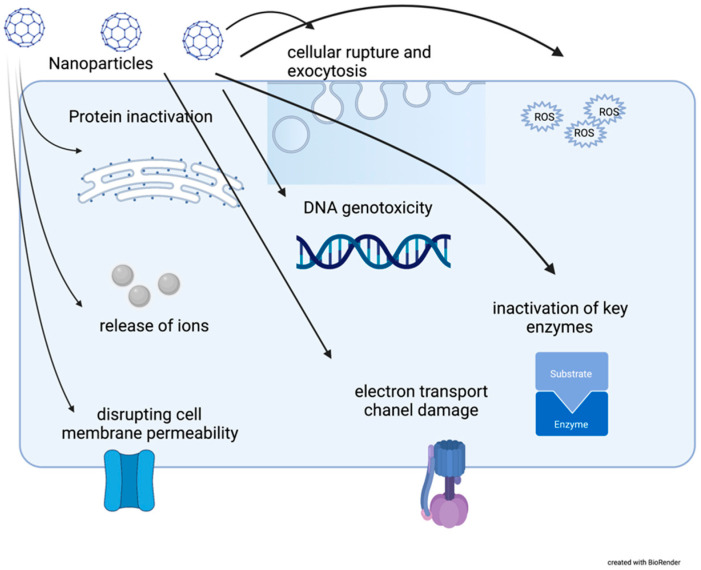
Nanoparticles’ mode of action on bacterial cells.

Metal NPs have been the most studied in relation to antimicrobial potency in wound healing. Among metal NMs, the most studied particles are silver NPs, yet metal oxide NPs such as zinc oxide, copper oxide and iron oxide also seem promising as antimicrobial treatments [15,16]. Due to their small surface area, NPs can penetrate biofilm and eventually penetrate to intracellular bacteria. The high surface area to volume ratio of nanoparticles allows drug loading, which can result in synergistic antibiofilm efficacy. NPs have antimicrobial properties thanks to oxidative stress, formation of reactive oxygen species, metal ion release and nonoxidative mechanisms, enzymatic inhibition, DNA damage and bacteria wall disruption [17,18,19] [Figure 2] [Table 1]. Inhibition of bacterial adhesion by NPs is a key mechanism that enables them to prevent biofilm formation. In comparison to antibiotics, NPs may infiltrate into the matrix, destroy the extracellular polymer substance (EPS) and eventually destroy the bacteria within the biofilm.

Owing to multiple modes of antimicrobial action of NPs, they have a high potential to reduce the prevalence of multiresistant bacteria in patients with chronic wounds [20,21]. Furthermore, they can also protect the drugs from enzymatic degradation in biofilm environment [22]. Therefore, they can be used alone if they have antimicrobial properties or as nanocarriers of antibiotics to help them reach therapeutic concentration in the infected tissue [23,24].

There is still, however, a lack of data on pharmacokinetics and pharmacodynamics of NPs therapy, especially in terms of clinical trials and possible applications separately and in combination therapy with antibiotics. Possible NPs resistance and adverse effects are other challenges for NPs that await to be discovered in order to implement NPs-based treatment options for the management of nonhealing infected wounds. 

This review’s aim is to describe in detail the mode of action of those molecules that have been proven to have an antimicrobial effect on biofilm and therefore help to eradicate bacteria from chronic wounds [25,26,27].

## 2. Presentation of NPs

### 2.1. Silver NPs

Silver NPs (Ag NPs) are the most widely used and known NPs, though their exact mode of action remains unknown [28]. Multiple mechanisms have been suggested as direct interaction with the bacterial membrane inhibiting cell wall synthesis or excavation leading to cell lysis [29,30,31]. Silver particles have been known for their intrinsic antibiofilm properties that are owing to their surface functional groups and ion release that can interact with biofilm.

It has been proven that Ag NPs have antibacterial activity due to continuous generation of Ag+ ions that release reactive oxygen species (ROS) [32]. Ag+ ions are bonded to thiol-containing proteins and inhibit those that also boost ROS production [33,34,35]. They also prevent the penetration of amines, thiols and carboxylates to biofilm [36]. Furthermore, they hasten the wound healing by downregulation of metalloproteinases, which belong to the collagenase enzyme group and are essential for wound healing. Their overexpression and therefore higher load leads to underexpression of key growth factors and fibronectin [37]. By lowering the metalloproteinase secretion and enhancing cell apoptosis, NPs regulate the inflammatory reaction in the wound bed and eventually shorten the first phase of the healing pathway. Moreover, NPs in wound dressings have been shown to control TNF-α expression that further shortens the inflammatory stage of wound healing, as they inhibit wound necrotization [38].

A study by Kalishwaralal et al., 2010 found that Ag NPs at a concentration of 100 nM prevented biofilm formation by *P. aeruginosa* and *S. epidermidis* by preventing bacterial adhesion to the surface [39]. Mohanty et al., 2012 confirmed that Ag NPs decreased *P. aeruginosa* biofilm by 65% and *S. aureus* biofilm by 88% [40]. Martinez-Gutierrez et al., 2013 showed that Ag NPs prevented the formation of *P. aeruginosa* biofilm and killed bacteria in already existing biofilm [41]. Another study showed that Ag NPs are also effective against Mycobacterium spp. biofilms [42]. 

Ag NPs can also boost the antibacterial effect of antibiotics. There are studies that support the synergistic effect between Ag NPs and aztreonam, ampicillin, kanamycin, streptomycin and vancomycin against *E. Coli* and *P. aeruginosa* [43]. Ag NPs with citrate and aztreonam showed antibiofilm efficacy against *P. aeruginosa*, whereas treatment with Ag NPs with ampicillin, oxacillin and penicillin inhibited and reduced MRSA biofilm by 94% [44,45].

Traditionally Ag NPs are synthesized by a chemical reduction process with the help of reducing agents in the presence of stabilizers in a suitable solvent. Recently, scientists have tried to find sustainable methods to prepare Ag NPs using plants, biological, or microbial agents as reducing and capping agents [46]. Ag NPs produced by green chemistry offer a new alternative for wound treatment without excessively polluting the environment. 

Gurunathan et al. synthesized Ag NPs with leaf extract of Allophylus cobbe. Those Ag NPs have been more effective against *P. aeruginosa* and *S. aureus* biofilm when combined with ampicillin and vancomycin then when using either antibiotics or NPs separately [47]. Other studies have shown that AgNPs with rhizome extract from Rhodiola rosea significantly inhibited *P. aeruginosa* and *E. Coli* biofilm formation [48].

Ferreres et al. manufactured new metal-enzyme NPs against biofilm, that consists of α-amylase and silver. Results are promising, as approximately 80% of *S. aureus* and *E. Coli* biofilm was eradicated [49]. 

### 2.2. Gold NPs

Gold NPs (Au NPs) as Ag NPs exhibit antibacterial and antibiofilm activity by interacting with sulfur-containing constituents in the cell membrane and leading to disruption of the cell wall [50,51,52]. Au NPs cause structural damage to the biofilm, kill sessile cells and mechanically disperse the cells in the suspension. Both Au and Ag NPs have catalytic activity as peroxidase, glucose oxidase and superoxide dismutase altogether [53]. This activity explains how they lead to oxidative stress in bacteria by increasing ROS production [54,55]. Moreover, positively charged NPs disrupt metabolic processes and lead to perforation and leakage through negatively charged bacterial membrane [56,57,58]. An additional advantage of Au NPs is that they inhibit intracellular ATP synthesis and tRNA binding [18,59]. Au NPs’ photothermal properties enable them to inhibit biofilm formation and ablate bacteria [60]. Au NPs synthesized with Mentha piperita (peppermint) have been effective against Gram negative *E. Coli* strains, but not against Gram positive *S. aureus* [61]. Au NPs produced by Euphorbia hirta have also showed inhibition of 88% of *E. Coli* strains, 86% of *P. aeruginosa* and 94% Klebsiella pneumonia [62]. Au NPs were also proven to be effective against Salmonella typhi and Enterococcus faecalis [63]. Au NPs also exhibit some antifungal properties [64,65,66]. 

Furthermore, gold is stable against oxidation, which makes it nontoxic. However, Au NPs are costly, difficult to store and do not have a high antibacterial spectrum [67]. 

### 2.3. Metal Oxide NPs

Metal oxide NPs such as iron oxide (Fe_3_O_4_), zinc oxide (ZnO), copper oxide (CuO), magnesium oxide (MgO) and titanium dioxide (TiO_2_) are known to have antibacterial properties over both Gram positive and Gram negative bacteria [68,69,70]. Their mode of action is based on ROS release through the Fenton reaction, intrinsic photocatalytic activity and the release of metallic ions [71,72].

Antimicrobial activity of NPs in general can be due to two main modes of action, namely the properties of the NPs themselves and the properties of the released metal ions, both of which can have a major influence on their antibacterial activity. The rate of dissolution is crucial for toxicity, as NPs with a higher rate of dissolution usually show increased toxicity. Similarly, the smaller the NPs and the higher their surface–volume ratio, the higher the toxicity towards bacteria they have, supposedly owing to their increased mobility [73]. Metal ions are the major reason for NPs’ toxicity towards bacteria, whereas NPs are helping to increase the metal ion concentration at the target place. 

As already mentioned, metal ion NPs may have a different mode of antibacterial action than the free metal. Some NPs as CuO or ZnO present bactericidal mechanisms that are far different than Cu or Zn properties. This has been proven as a nonredox molecule may become redox-active as NPs and catalyze ROS production [74,75]. Earlier named CuO and ZnO NPs are able to produce ROS outside the cell and lead to cell damage by lipid peroxidation. Small NPs then can pass inside the cells and may further influence the bacterial DNA/RNA, protein, carbohydrate, lipids or ATP production and modifications.

Wound management is one of the most innovative fields of medicine, where ongoing research is revolutionizing the field every day. There are numerous wound dressings that are made of nanofibers, hydrogels, hydrocolloids, alginates, gels and foams that differ from one another by their mode of action on the wound bed. Metal oxide NPs can be coated into wound dressings made of polyvinyl alcohol, chitosan, polycaprolactone or cellulose [76,77]. These dressings not only enhance the antibacterial effect of NPs, but also accelerate wound healing. These dressings would be a promising alternative for conventional treatment.

#### 2.3.1. Zinc Oxide NPs

ZnO NPs are recognized as safe by the US Food and Drug Administration (21 CFR 182.8991) [78]. Their properties have been carefully studied, as they show the highest toxicity against multiresistant bacteria [79,80]. Their toxicity does not only depend on destroying bacterial cell wall and bacterial death, yet they also liberate ROS in the biofilm microenvironment and induce their solubility [81,82,83,84]. 

Zn is commonly used in cosmetics and pharmaceutical products due to its antimicrobial, anti-inflammatory and hygroscopic properties. It has collagenolytic activity and reduces necrotic material and annihilates infections in the wound bed, stimulates epithelialization and hastens complete wound closure. ZnO NPs applied in Unna boots have been shown to reduce inflammation and reduce wound size [85]. Furthermore, another interesting mode of action of ZnO NPs is inhibition of bacterial kinase that, in a simple way, leads to bacteria apoptosis. 

Azam et al. compared the antibacterial effects of ZnO, CuO and Fe_2_O_3_ NPs and showed that ZnO NPs are the most effective against Gram positive *S. aureus*, Bacillus subtilis and Gram negative *E. coli*, *P. aerogenosa*. ZnO NPs had a maximum inhibitory effect at relatively low concentration. Another experiment of Azam et al. proved that ZnO inhibited 72% more *E. Coli*, 80% *S. aureus*, 88% *P. aeruginosa* and 84% of *B. subtilis* than CuO or Fe_2_O_3_. A study by Beak and Wang et al. supported the initial results of Azam et al. [86]. 

#### 2.3.2. Iron Oxide NPs

Aside from ROS generation through the Fenton reaction, iron oxide NPs exhibit magnetic properties [87]. By electrostatic interaction between positive NPs and negatively charged bacteria, drug resistant *S. aureus* and *E. Coli* can be trapped. Thereafter, bacteria are killed by a radiofrequency current owing to the loss of membrane potential and disruption in membrane channels [88]. Iron oxide NPs, through the Fenton reaction, produce free radicals that degrade EPS and kill bacteria within biofilm. 

Another study showed that the iron oxide NPs coating nisin, activated by both electric and electromagnetic fields, increased the antibacterial efficiency of nisin that is known to be inefficient against Gram negative bacteria. Activation of NPs led to increased permeability and local hyperthermia and resulted in synergistic antimicrobial effects on both Gram positive Bacillus subtilis and Gram negative *E. Coli* [89]. 

Iron oxide NPs exhibit peroxidase-like activity only at specific pH characteristics, for example for *S. mutans*, so that healthy tissues are spared [70,90]. 

#### 2.3.3. Magnetite NPs

In addition to other metal oxide NPs, Fe3O4 does not show any antibacterial effect, yet it has no cytotoxic effect and has superparamagnetic properties, which makes it an ideal nanocarrier [91]. Drugs that are carried with such NPs can target the biofilm at highest concentration. Due to positive charge and large surface area, it can cause mechanical disruption of the negatively charged bacterial wall [92,93,94]. 

Magnetite NPs boost the action of antibiotics against biofilm, especially penicillin, streptomycin, erythromycin, kanamycin and cefotaxime against *S. aureus* and amphotericin, and nystatin against Candida spp. biofilms has been recorded [95]. Furthermore, oleic acid magnetite NPs inhibited abundance of *S. aureus*, Saccharomyces cerevisiae, *C. tropicalis*, *C. albicans*, *C. famata*, *C. krusei* and *C. glabrata* [83]. 

Scientists are trying to combine naturally abundant plants with antibacterial properties and NPs. In a study where Rosmarinus officinalis oil and magnetite NPs were coated on prosthetic devices, Candida albicans biofilm was significantly reduced to approximately 2% within 72 h [96]. 

NPs with the use of chitosan and polypyrrole, which is a conductive polymer, were proven to inhibit formation of biofilm of *P. aeruginosa*. This particle directly inhibited the formation of virulence factors such as pyocyanine, rhamnolipids and pyroverdine and inhibited motility of bacteria [97]. 

#### 2.3.4. Titanium Dioxide NPs

For a long time, titanium dioxide has been known for its antimicrobial activity and is therefore widely used as a disinfecting ingredient in cosmetics. It has been shown to be bactericidal against both Gram positive and Gram negative bacteria, making it a perfect compound for wound treatment as those are usually infected with a mix of bacteria [98].

Titanium surfaces coated with TiO_2_ nanotubes by anodization route boosted antibacterial properties [99,100,101]. Recent studies showed a 70% decrease in bacterial adhesion after 90 min incubation and significant inhibition of biofilm formation after 24 h of incubation [102,103]. Such titanium surfaces significantly disrupt bacterial adhesion, mainly by inhibition of surface topography [104]. 

Several studies have shown that titanium surfaces reduce inflammation, accelerate bone regeneration and help platelet adhesion and activation, which makes them a promising agent in wound healing [105,106,107,108]. Addition of TiO_2_ reduced inflammation and swelling around the wound site and increased thermal stability while decreasing the scaffold pore size of the material [109]. 

### 2.4. Carbon NPs

Carbon can be an ingredient of organic nanomaterials (NMs), as it has shown diverse properties. Carbon dot is a term for various carbon NMs such as polymers, rods, sheets, fullerenes and graphene [110]. Carbon-based NMs can be synthesized separately or ennobled with metal based NMs. 

#### 2.4.1. Carbon Nanotubes

Carbon nanotubes (CNT) have an incredibly large surface area which allows high drug load and adsorption both inside the tube and on the outer surface. This eventually helps to overcome bacterial resistance mechanisms such as multidrug efflux pumps or permeability regulation and protects the antimicrobial substance from pH or enzymatic degradation. CNTs are promising since they can stabilize the drug by encapsulation and reduce the drug’s toxicity by deacceleration of its release [111]. Nanocarriers can help antibacterial agents penetrate through biofilm, reduce biofilm abundance and bacteria viability.

Isoniazid loaded chitosan+CNTs hastened the wound healing in guinea pigs with secondary bone tuberculosis initially infected subcutaneously with Mycobacterium tuberculosis. Secondary infections are even more difficult to treat than primary wound infections [112]. Those NMs were reported to decrease CD3+ and CD4+ T cell count and eventually muted the immunological response. Scientists reported 94.6% higher relative reduction in ulcers’ size than with isoniazid alone, which directly implies that CNTs might boost the response of several antimicrobial agents compared to the same antimicrobials used alone. 

Another study’s results have shown that functionalized multiwall CNTs in polyvinyl alcohol conjugated with glucose oxidase express antibacterial properties due to generation of hydrogen peroxide from the oxidase [113]. Furthermore, it is worth noticing that CNTs yield increased wound healing rate by their ability to promote cell migration when embedded in hydrogels. The latter is widely used to produce wound dressings thanks to their biocompatibility, mechanical rigidness and hydrophilicity. A recent study testing multiwall carboxylic functionalized CNTs in fibrous hydrogels showed better healing outcomes than pure hydrogel. CNTs are thought to enhance adhesion of, for example, fibroblasts that might migrate and yield granulation of the wound bed, leading to hastened wound closure [114]. 

#### 2.4.2. Graphene

Recently, graphene-based NMs have been studied for their ability to shorten wound healing time and control the local infection in the wound bed. Graphene is an allotrope of carbon that consists of a single layer of atoms arranged in a two-dimensional honeycomb lattice that gives it a large surface area [115]. Graphene is the strongest known material, harder than diamond yet more elastic than rubber. Graphene as other carbon derivatives can also be incorporated in hydrogels to facilitate better cellular adhesion and differentialization. Due to the fact that graphene can maintain moisture within other environments, it is widely used for the production of drug carriers, biosensors, microelectromechanical systems (MEMS), biomimetic micro-and nanorobots and microfluidic devices [116]. Graphene-bearing Ag NPs have shown synergistic effects of both antimicrobial compounds in shortening wound healing time [117]. Furthermore, graphene can act as a photocatalyst and photodegrading agent in daylight and cleave biofilm polysaccharide linkages, disrupt biofilm and kill bacteria within it.

#### 2.4.3. Carbon Quantum Dots

Carbon quantum dots (CQD) are the newest invention from carbon-based NMs. This group stands for zero-dimensional carbon materials. They have been proven to have antibacterial and, most interestingly, antibiofilm properties. Their role in wound healing is mainly worth noting as reducing the inflammation and promoting collagen deposition, granulation tissue development [110]. A study on CQDs embedded in chitosan dextran hydrogel showed promising results that might encourage scientists to develop new formulas with CQD and test their pharmacokinetics and bioavailability [118]. 

### 2.5. Mesoporous Silica NPs 

Mesoporous silica NPs (MSNPs) are used as vehicles for antibacterial agents such as antibiotics. The main advantages of MSNPs are their high loading capacity, simple production, biocompatibility, high degree of tunability, morphology and pore diameter. They are able to shield the active particle and increase its bioavailability. Furthermore, they enable sustained release of the active substance, which helps to maintain an optimum drug level in the bloodstream. What makes them special is that they can be produced in big quantities and in a wide variety of morphologies using diverse strategies [119,120]. In this sense, they can carry more than one active substance simultaneously and act as a combined therapy. 

MSNPs carrying levofloxacin showed high efficacy and high penetration within *S. aureus* biofilm, destroying it almost completely [121]. Bacterial biofilm is known to be a physical barrier for antibiotic penetration through the bacterial wall. That is why scientists are working on developing systems that enable antibacterial substances to penetrate it. MSNPs with levofloxacin and coated with concanavalin A were invented. Concanavalin A increases the efficiency of antimicrobial agents to internalize into the biofilm and eventually penetrate the mesopores and kill bacteria. Furthermore, this treatment option has been shown to have less side effects than conventional treatment for biofilm infections [122]. In another study, MSNPs with levofloxacin were grafted with third generation polycationic polypropylene imine dendrimer to provide the capability to interact with the bacterial membrane. Results confirmed that this combination provides an efficient antibiofilm effect on Gram negative bacteria [123]. 

## 3. Conclusions

Chronic wounds are a challenge for physicians worldwide, as infections of the wound bed are more and more difficult to eradicate due to antibiotic-resistant bacterial strains. 

NPs seem to be a promising treatment option for infection management, which is essential for the final stage of wound healing, which is complete wound closure. NPs can enhance an antibiotic’s mode of action by increasing its solubility and easing its transport into the cells or can be directly bacteriostatic or bactericidal. Research suggests that NPs might either protect conventional antibiotics from degradation by pH or enzymatic activity of biofilm microenvironment or decrease biofilms resistance to the eradication. There are no data on storage, administration and mucous interaction, as well as blood clearance and long-term results, safety and side effects. Undoubtedly, randomized controlled in vivo trials are needed to state the efficiency and place of NPs within novel medical treatment. 

## Figures and Tables

**Table 1:** Baseline characteristics of chosen nanoparticles and nanomaterials, that are described further in the article.

Nanoparticles/Nanomaterials	Mode of Action	Synthesis Methods	Possible Side Effects
Silver NPs	ROS generation, lipid peroxidation, inhibition of cytochromes of ETC, cell wall disruption, inhibition of cell wall synthesis, increase in membrane permeability, disruption of proton gradient resulting in lysis, adhesion to cell surface causing lipid and protein damage, ribosome destabilization, damaging DNA, disruption of biofilms	laser ablation, gamma irradiation, electron irradiation, chemical reduction, photochemical methods, microwave processing and biological synthetic methods, such as extracts from *Artemisia cappilaris*, extract from aloe vera, extract from *Acalypha indica.,* leaf extracts from *Rhizopus oryzae,* extracts of *Cocus nucifera*	Might be toxic towards keratinocytes and fibroblasts. More resistance towards AgNPs due to genetic modifications in bacteria. Ag NPs can deposit in liver, spleen, lungs and other organs and result in their dysfunction.
Gold NPs	Loss of membrane potential, disruption of respiratory chain, reduced ATPase activity, decline in subunit of ribosome for tRNA binding, bacterial membrane disruption	chemical, thermal, electrochemical and sonochemical pathways reduction by agents, biological methods using different bioreductant and capping agents such as terpenoids, phenolic compounds, proteins, polysaccharides and nicotinamide adenine dinucleotide (NAD from Citrullus lanatusrind from Plumbago zeylanica	Huge costs of production, alternative production methods should be searched, cost effectiveness is not known.
Metal oxide NPs	ROS production, disruption of membrane, adsorption to cell surface, lipids and protein damage, inhibition of microbial biofilm formation, DNA degradation, antioxidant activity	Chemical polyol method, microemulsions, thermal decomposition, electrochemical synthesis. Physical methods: plasma, chemical vapor deposition, microwave irradiation, pulser laser method, sonochemical reduction, gamma radiation, biological methods using extracts from *Caltropis procera* fruits or leaves, leaf extract of lemongrass	The high toxicity of CuO NPs causes oxidative lesions, while ZnO and TiO_2_ can cause DNA damage.
Carbon nanomaterials	Inhibition of bacterial adhesion, cell membrane damage, leakage of cytoplasmic contents, higher oxygen consumption rate Graphene ROS protein dysfunction, oxidative stress, laddering of DNA, membrane damage, disturbance of the membrane permeability	Carbon nanomaterials arc discharge, laser ablation, chemical vapor deposition (CVD), ball milling, the flame procedure, solution mixing Graphene chemical reducing factors, thermal baking, photoreduction and microwave-assisted reduction	Insoluble in most solvents, might be toxic.
Mesoporous silica NPs	Inhibition of adhesion onto surfaces and thus the prevention of biofilm formation, physical damage to cell membranes, ROS production and endolysosomal burden. Mostly used as nanocarriers as they increase drug solubility, pharmakinetics and pharmadynamics, also reducing systemical toxicity.	Sol–gel process, reverse microemulsion and flame synthesis.	Toxicity, protein fouling and immunogenicity are possible.

## Data Availability

The data presented in this study are available on request from the corresponding author.
